# Investigation of morpho-physiolgical traits and gene expression in barley under nitrogen deficiency

**DOI:** 10.1038/s41598-024-59714-z

**Published:** 2024-04-17

**Authors:** Zohreh Hajibarat, Abbas Saidi, Habibollah Ghazvini, Zahra Hajibarat

**Affiliations:** 1 Department of Cell and Molecular Biology, Faculty of Life Sciences and Biotechnology, Shahid Beheshti University, Tehran, Iran; 2 Seed and Plant Improvement Institute, Agricultural Research, Education and Extension Organization (AREEO), P.O. Box 31587-77871, Karaj, Iran

**Keywords:** Barley, Correlation, Gene expression, Nitrogen deficiency, Physiological traits, Plant sciences, Environmental sciences

## Abstract

Nitrogen (N) is an essential element for plant growth, and its deficiency influences plants at several physiological and gene expression levels. Barley (*Hordeum vulgare*) is one of the most important food grains from the *Poaceae* family and one of the most important staple food crops. However, the seed yield is limited by a number of stresses, the most important of which is the insufficient use of N. Thus, there is a need to develop N-use effective cultivars. In this study, comparative physiological and molecular analyses were performed using leaf and root tissues from 10 locally grown barley cultivars. The expression levels of nitrate transporters, *HvNRT2* genes, were analyzed in the leaf and root tissues of N-deficient (ND) treatments of barley cultivars after 7 and 14 days following ND treatment as compared to the normal condition. Based on the correlation between the traits, root length (RL) had a positive and highly significant correlation with fresh leaf weight (FLW) and ascorbate peroxidase (APX) concentration in roots, indicating a direct root and leaf relationship with the plant development under ND. From the physiological aspects, ND enhanced carotenoids, chlorophylls a/b (Chla/b), total chlorophyll (TCH), leaf antioxidant enzymes such as ascorbate peroxidase (APX), peroxidase (POD), and catalase (CAT), and root antioxidant enzymes (APX and POD) in the Sahra cultivar. The expression levels of *HvNRT2.1*,* HvNRT2.2*, and *HvNRT2.4* genes were up-regulated under ND conditions. For the morphological traits, ND maintained root dry weight among the cultivars, except for Sahra. Among the studied cultivars, Sahra responded well to ND stress, making it a suitable candidate for barely improvement programs. These findings may help to better understand the mechanism of ND tolerance and thus lead to the development of cultivars with improved nitrogen use efficiency (NUE) in barley.

## Introduction

Nitrogen (N) is one of the most important components of biomolecules, amino acids, nucleotides, proteins, chlorophyll, and many plant hormones, as well as an essential component for plant growth and development. Regulating leaf photosynthesis is a crucial role of nitrogen (N) as a mineral nutrient^1^. The global production volume of barley amounted to about 150.48 million metric tons in the 2022/2023 cropping season year, increasing from around 145.93 million metric tons in 2021/2022^2^. N deficiency (ND) negatively affects leaf chlorosis, bud growth, and overall plant growth^3^. The deficiency of this essential element causes nutrient imbalance affecting several metabolic pathways including the increased production of reactive oxygen species (ROS)^4,5^. In rice, ND and the antioxidant system resulted in decreased light-harvesting capacity and increased thermal dissipation of absorbed energy^6^. An increase in ROS imposes oxidative stress on plants, which utilize antioxidant enzymes, such as ascorbate oxidase (APX), peroxidases (POX), and catalase (CAT), to prevent excessive ROS accumulation^7^. Wheat genotypes responded differentially to N supply in relation to leaf growth and photosynthesis as well as the maintenance of metabolic constituents^8^. At the early vegetative stage of plant life, ND adversely influences crop yield, which cannot be offset by N application at later stages^9^. Nitrogen fertilizer is applied to enhance crop yield because its availability strongly affects crop productivity. A significant amount of N contaminates ground and surface water and emits the greenhouse gas, nitrous oxide^10^. Thus, crop genotype development with improved nitrogen use efficiency (NUE) can aid in sustainable agriculture and high productivity under low-input conditions. NUE is a complex trait involving physiological, developmental, and environmental factors. This includes the plant's ability to absorb, transport, and remobilize N in the soil. Considerable efforts have been made to understand the molecular basis of plant response to N and to detect N-responsive genes due to NUE complexity^11^.

Previously, relative comparisons were made for N deficiency tolerance (NDT) traits (shoot and root biomass, plant height, root length, and chlorophyll content) under low N and control conditions. These traits were considered the selection criteria for identifying rice genotypes with improved adaptability^12^. N uptake by plants is mainly associated with nitrate transporters (NRTs). For instance, of seven NRT2s identified in *Arabidopsis thaliana*^13^, AtNRT2.1, AtNRT2.2, AtNRT2.4, and AtNRT2.5 are responsible for approximately 95% of total NO^-^_3_ uptake under N-limited conditions^14,15^. Four *NRT2* genes were found in rice^16^. To provide N sources, high-tension transporters NRT2 and NRT3 play important roles in N uptake^13^. The role of NRT2.1 in the high-affinity NO^−^ 3 (HATs) transport system has been proved in some studies^16,17^. The NRT2 transporters have a high affinity for NO^-^_3_ and are induced under NO^-^_3_-limiting conditions^18^. However, a major limitation is only using a single genotype in most of the studies. Barley, a robust cereal crop grown in a wide range of agricultural settings from highly developed to subsistence environments, ranks fourth in global importance behind wheat, maize, and rice^19^. To develop N-effective barely genotypes, it is necessary to identify candidate genes having a critical role in NUE. In the present study, traits were investigated in 10 different varieties of barley to identify candidate genes and molecular mechanisms involved in N. In addition, the physiological and morphological traits of these cultivars were evaluated in response to ND stress. The expression of genes related to NO^-^_3_ metabolism in leaves and roots was analyzed under NO^-^_3_-limiting conditions to highlight the correlation between the traits and gene expression related to N metabolism.

## Results

### Morphological traits under ND conditions

Among the morphological traits measured 7 and 14 days after applying ND, the Sahra cultivar showed a significant increase in dry root weight (DRW) 7 days after ND application as compared to normal conditions. However, the Noroz cultivar showed a significant decrease in DRW 7 and 14 days after ND application as compared to normal conditions. After 14 days of ND application, Noroz showed a significant decrease in fresh root weight (FRW) (Table 1).

**Table 1:** Comparison of means for morphological traits [root dry weight (RDW), leaf dry weight (LDW), root fresh weight (RFW), and leaf fresh weight (LFW)] measured in 10 barley cultivars.

Cultivars	Treatments	RDW	LDW	RFW	LFW
Sahra	N7	11.8 de	17.95 bcdef	124.35 bcdefg	223.85 abcd
Sahra	NC7	23.8 ab	15.85 def	106.1 cdefg	219.95abcd
Sahra	N14	12.75 b	27.95 ab	44.85 abcdef	224.2 a
Sahra	NC14	18.8 b	33.9 ab	29.3 bcdef	231.25 abc
Noroz	N7	9.2 ef	34.7 a	225.4 a	337.15 d
Noroz	NC7	19.35 abcd	24.8 abcde	164.75 abcde	278 bcd
Noroz	N14	43.8 a	28.85 ab	62.45abcd	291.55ab
Noroz	NC14	7.75b	23.1 ab	8.05f.	221.65 ab
Armaghan	N7	17.45 bcde	15.55 def	114.65 cdefg	137.05 ab
Armaghan	NC7	13.9 cdefg	19.73 bcdef	210.833 ab	190.97 ab
Armaghan	N14	21.9 b	22.05ab	28.2cdef	176.35ab
Armaghan	NC14	15.4 b	33.5ab	27.4cdef	255.5abcd
Yousef	N7	16.7 bcdef	24.8 abcde	187.9 abc	296.1 abcd
Yousef	NC7	15.85 bcdefg	21.5 bcdef	185.35 abc	290.85 abc
Yousef	N14	10.83 b	25.8 ab	29.6 bcdef	261.3 abcd
Yousef	NC14	10.3 b	23.75 ab	13.5 f.	218.35 abcd
Nobahar	N7	16.3 bcdefg	15.55 def	129.5 bcdefg	296.95 cd
Nobahar	NC7	18.85 abcd	23.65 abcdef	150.75 abcdef	290.45 abcd
Nobahar	N14	22.7 b	31.6 ab	57.45 abcde	234.9 abcd
Nobahar	NC14	10.5 b	31.15 ab	18.55 ef	212 abcd
Oxin	N7	15 bcdefg	23.2 bcdef	131.5 bcdefg	291.15 bcd
Oxin	NC7	19.25 abcd	25.35 abcd	130.95 bcdefg	268.5 abcd
Oxin	N14	9.95 b	23.85 ab	18.6 ef	257.3 ab
Oxin	NC14	16.25 b	29.65 ab	27.95 cdef	219 abcd
Zehak	N7	14.85 bcdefg	16.5 cdef	114.55 cdefg	220.25 bcd
Zehak	NC7	12.25 defg	21.8 bcdef	134.9 bcdefg	285.4 abcd
Zehak	N14	8.5 b	31.6 ab	28.85 bcdef	299.9 bcd
Zehak	NC14	9.55 b	26.7 ab	15.7 ef	262.9 abcd
Nimroz	N7	18.9 abcd	15.95 def	16.9 abcde	225.5 abcd
Nimroz	NC7	16.45 b	22.75 ab	20.45 def	224.65 abc
Nimroz	N14	12.9 b	18.6 b	13.4 f.	133.45 cd
Nimroz	NC14	12.9b	18.6b	13.4f.	133.45d
Khatam	N7	8.7 efg	13.05 f.	68.6 efg	172.85bcd
Khatam	NC7	13.7cdefg	19.56 bcdef	122.4 bcdefg	238.95 bcd
Khatam	N14	13.6 b	23.2 ab	15.65 ef	187.85 abcd
Khatam	NC14	10.8 b	22.4 ab	14.85 ef	149.15 cd
Goharan	N7	8.3 efg	28.3 ab	118.8 cdefg	297.3 ab
Goharan	NC7	7.7 fg	20.05 bcdef	95.65 defg	259.5 abcd
Goharan	N14	11.55 b	27.3 ab	77.6 a	285.96 ab
Goharan	NC14	10.8 b	24.45 ab	40.1 abcdef	261.4abc

N7, normal at 7 days after stress; N14, normal at 7 days after stress; NC7, stress at 7 days after stress;

NC14, stress at 7 days after stress.

### Physiological traits under ND conditions

#### Chlorophylls a/b

**Figure 1:**
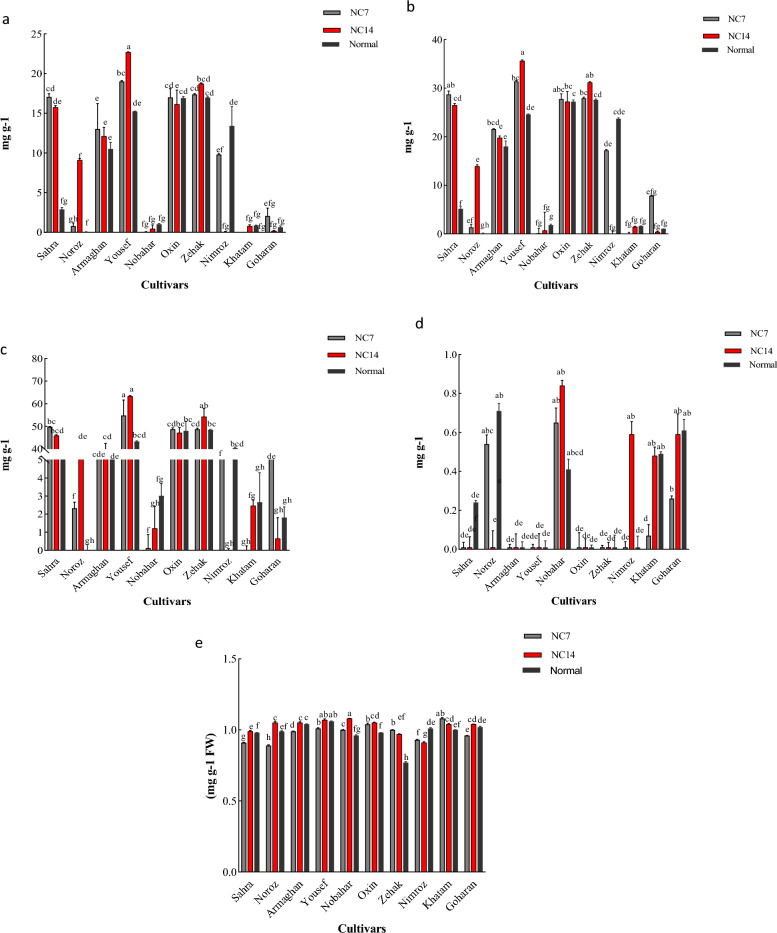
The effects of ND on 10 barley cultivars for chlorophyll a (**a**), chlorophyll b (**b**), total chlorophyll (**c**), carotenoid (**d**), and protein content (**e**) at 7 and 14 days stress periods. Values represent means of three replications per treatment. Different letters demonstrate significant differences between treatments (*P* < 0.05, Duncan’s Multiple Range Test). NC7:7 days after ND application; NC14: 14 days after ND application.

The Sahra and Yousef cultivars showed a significant increase in the chlorophyll a (Ch-a) content 7 and 14 days after the ND application (Fig. 1a). Ch-b content increased significantly in the Sahra and Yousef cultivars 7 and 14 days after the ND application. When compared with normal conditions, the Armaghan cultivar showed a significant increase in Ch-b only 7 days after the ND application (Fig. 1b).

#### Total chlorophyll content

Total chlorophyll (TCH) content increased significantly in the Sahra and Yousef cultivars 7 and 14 days after the ND application as compared to normal conditions. The Nimroz cultivar showed a significant decrease in TCH content at both time points after the ND application. In the Goharan cultivar, a significant increase in TCH content was observed only 7 days after the ND application (Fig. 1c).

#### Carotenoid content

The Khtam cultivar showed a significant decrease in carotenoid content 7 days after the ND application whereas the Nimroz cultivar showed a significant increase in carotenoid content when compared to normal conditions 14 days after the ND application (Fig. 1d).

#### Protein content

The Zehak, Nobahar, Khatam, and Oxin cultivars showed significant increases in protein content 7 and 14 days after the ND application. As compared to normal conditions, the Sahra cultivar showed a significant increase in protein content 14 days after the ND application. The Nimroz cultivar showed a significant decrease in protein content 7 and 14 days after the ND application as compared to normal treatment (Fig. 1e).

#### APX content in leaves

**Figure 2:**
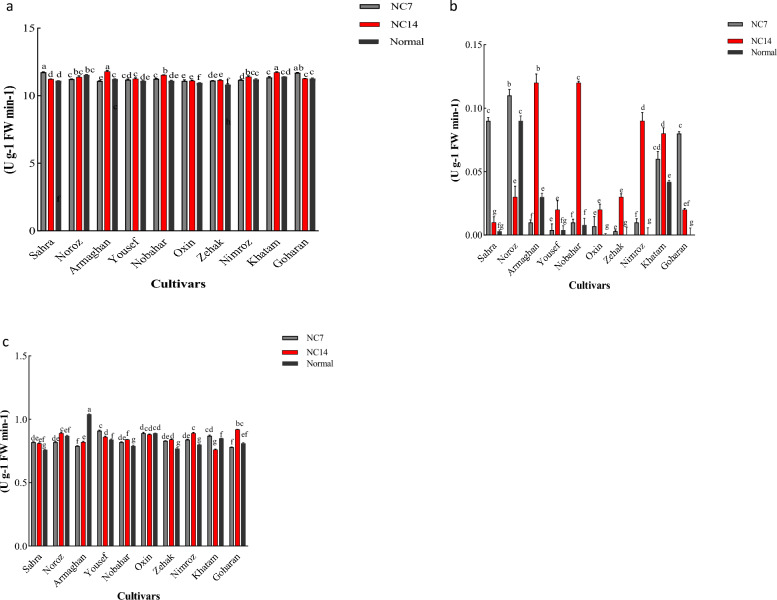
The effects of ND on APX (**a**), CAT (**b**), and POD (**c**) antioxidative enzyme activities in shoot of barley cultivars under different concentrations. The error bars (mean ± SE) followed by various letters are statistically significant (*P* < 0.05, Duncan’s Multiple Range Test). Significant differences between the two concentrations are marked with different letters. NC7:7 days after ND application; NC14: 14 days after ND application.

The analysis of the APX enzyme in the leaves of Sahra and Goharan cultivars showed a significant increase 14 days after the ND application. The Nobahar, Oxin, and Zehak cultivars showed a significant increase 7 and 14 days after applying ND compared to the normal treatment (Fig. 2a).

#### CAT content in leaves

The Yousef, Oxin, Khatm, and Goharan cultivars showed significant increases in the CAT content of leaves 7 and 14 days after the ND application as compared to the normal treatment. The CAT content increased significantly in the Sahra cultivar 7 days after the ND application. Further, a significant decrease occurred in the CAT content 14 days after the ND application while a significant increase was observed in the Noroz cultivar 7 days after the ND application (Fig. 2b).

#### POD content in leaves

The POD enzyme showed significant differences in response to the ND application in different cultivars at the two times. The Yousef, Nimroz, Nobahar, Zahak, and Sahra cultivars showed significant increases in the POD content 7 and 14 days after the ND application as compared to the normal treatment. After 14 days of the ND application, POD content significantly increased in Noroz and Goharan cultivars as compared to the normal treatment. In the Armaghan cultivar, a significant decrease was observed 7 and 14 days after the ND application (Fig. 2c).

#### APX content in roots

**Figure 3:**
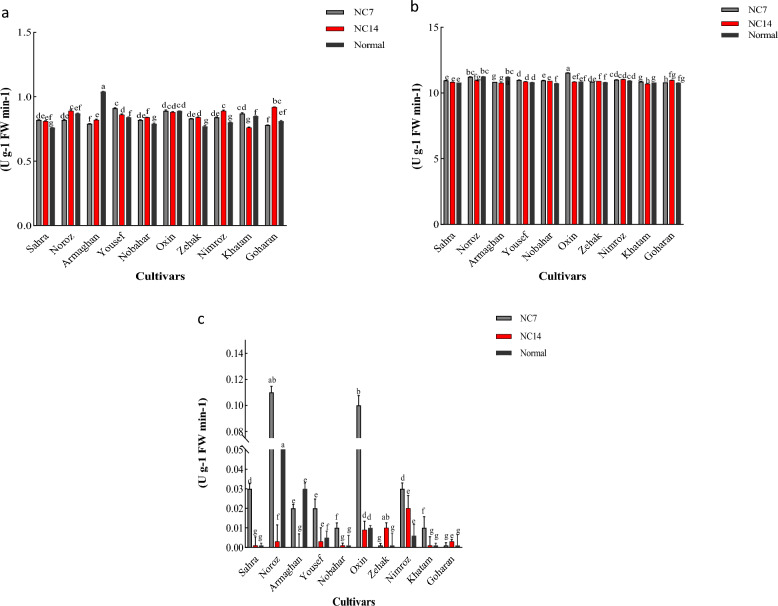
The effects of ND on APX (**a**), CAT (**b**), and POD (**c**) antioxidative enzyme activities in root of barley cultivars under different concentrations. The error bars (mean ± SE) followed by various letters are statistically significant (*P* < 0.05, Duncan’s Multiple Range Test). Significant differences between the two concentrations are marked with different letter. NC7:7 days after ND application; NC14: 14 days after ND application.

The analysis of physiological traits in response to ND showed that the APX enzyme significantly varied in the roots 7 and 14 days after the ND application. The Sahra, Yousef, Nobahar, Zehak, and Nimroz cultivars showed significant increases in the APX content 7 and 14 days after the ND application (Fig. 3a).

#### CAT content in roots

The analysis of CAT content in response to ND showed that Yousef, Sahra, and Nimroz cultivars were not significantly different between the two ND application times. In the Oxin cultivar, a significant increase in the CAT content was observed 7 days after the ND application. In the Nobahar cultivar, CAT content increased significantly 7 and 14 days after the ND application. Zehak and Goharan showed a significant decrease 7 days after the ND application (Fig. 3b).

#### POD content in roots

The analysis of the POD enzyme in response to ND showed significant differences among 10 barley cultivars. The Sahra, Nimroz, Noroz, Oxin, Yousef, Khatam, and Nobahar cultivars showed a significant increase in the POD content 7 days after the ND application. Armaghan showed a significant decrease in POD content 14 days after the ND application (Fig. 3c).

#### The *HvNRT2* gene expression profile in shoots in response to ND

**Figure 4:**
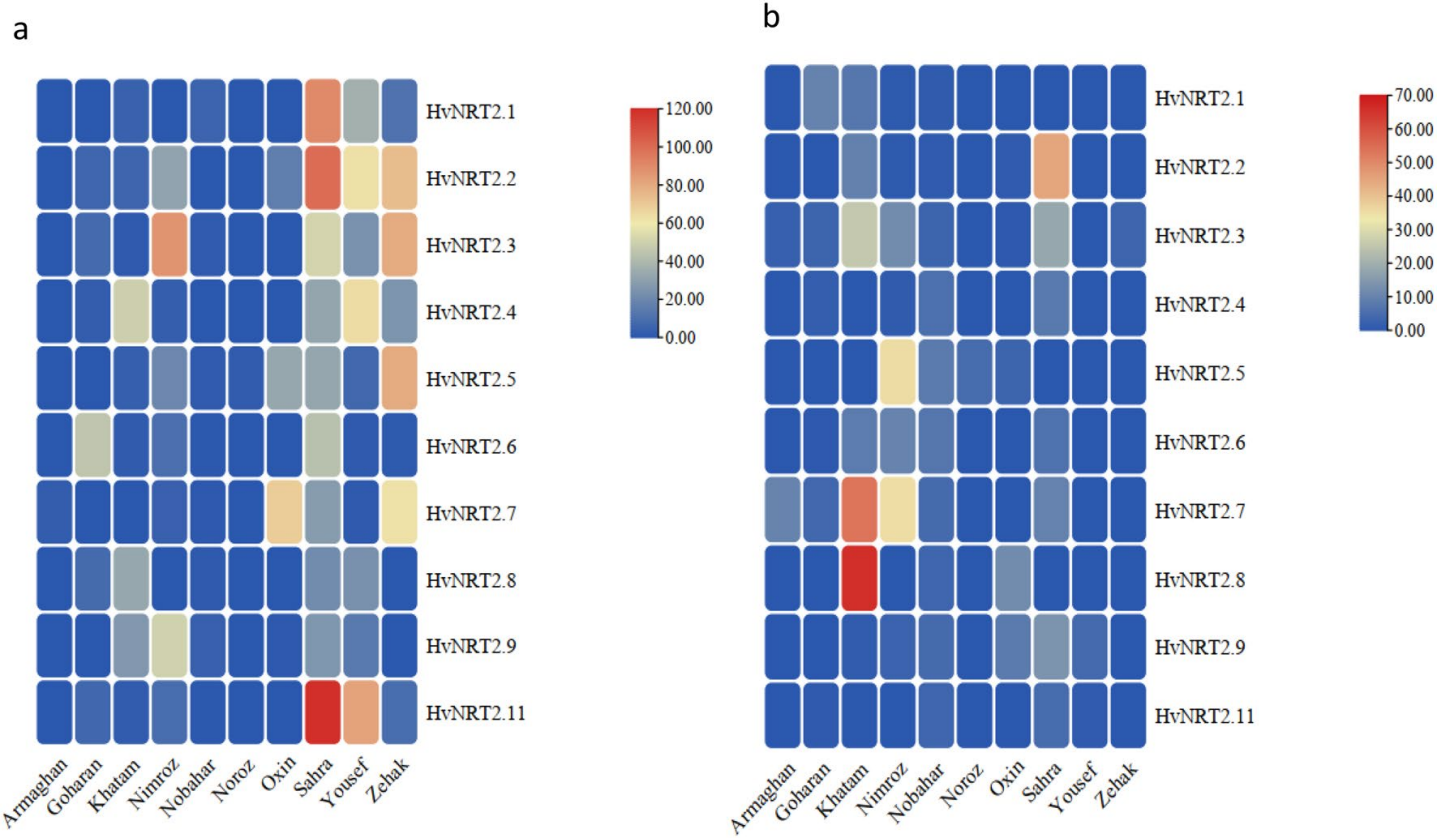
Heatmaps representing the expression profiles of leaf *HvNRT2* genes in response to ND, and their gene expression at 7 (**a**) and 14 (**b**) days after ND application for leaf. The heat map was generated using TBtools.

The expression of 10 genes in response to ND showed that the most expressed genes in shoots belonged to Sahra, Zehak, and Yousef cultivars 7 and 14 days after the ND application. In the Sahra cultivar, all *HvNRT2* genes were significantly increased 7 days after applying ND, as compared to 14 days after the ND application. In the Zehak cultivar*,* the *HvNRT2.1, HvNRT2.2, HvNRT2.3, HvNRT2.4, HvNRT2.5, HvNRT2.7*, and *HvNRT2.10* genes showed a higher significant expression after 7 days than 14 days after the ND application. In the Yousef cultivar, the *HvNRT2.1, HvNRT2.2, HvNRT2.3, HvNRT2.4, HvNRT2.5, HvNRT2.8, HvNRT2.9,* and *HvNRT2.11* genes revealed a higher significant expression 7 days after the ND application than 14 days after this treatment (Fig. 4). The Noroz cultivar showed an up-regulation of the *HvNRT2.5* gene 14 days after the ND application. In the Oxin cultivar, the *HvNRT2.2, HvNRT2.5,* and *HvNRT2.7* genes revealed a higher significant expression 7 days after applying ND than after 14 days. After 14 days of applying ND, the expression levels of *HvNRT2.8* and *HvNRT2.9* genes increased as compared to 7 days after the ND application. The 14-day ND application led to an increase in the expression levels of *HvNRT2.4* and *HvNRT2.5* genes in the Nobahar cultivar.

In the Nimroz cultivar, the *HvNRT2.2, HvNRT2.3, HvNRT2.9,* and *HvNRT2.11* genes showed a significantly higher expression 7 days after the ND application than after 14 days. The expression of *HvNRT2.*5 and *HvNRT2.7* genes increased in the Nimroz cultivar after 14 days as compared to 7 days after the ND application (Fig. 4). In the Khatam cultivar, the *HvNRT2.4* and *HvNRT2.9* genes were significantly expressed after 7 days as compared to the 14-day ND application. The expression of *HvNRT2.3, HvNRT2.7*, and *HvNRT2.8* genes indicated significantly higher levels 14 days after applying the ND than after 7 days. In the Goharan cultivar, the *HvNRT2.6* gene showed a significantly higher expression 7 days after applying ND than after 14 days. A significantly higher expression was observed in the *HvNRT2.1* gene 14 days after the ND application than after 7 days. In the Armaghan cultivar, the *HvNRT2.7* gene showed a significantly higher expression after 14 days than the 7-day ND application (Fig. 4).

#### The *HvNRT2* gene expression profile in roots in response to ND

**Figure 5:**
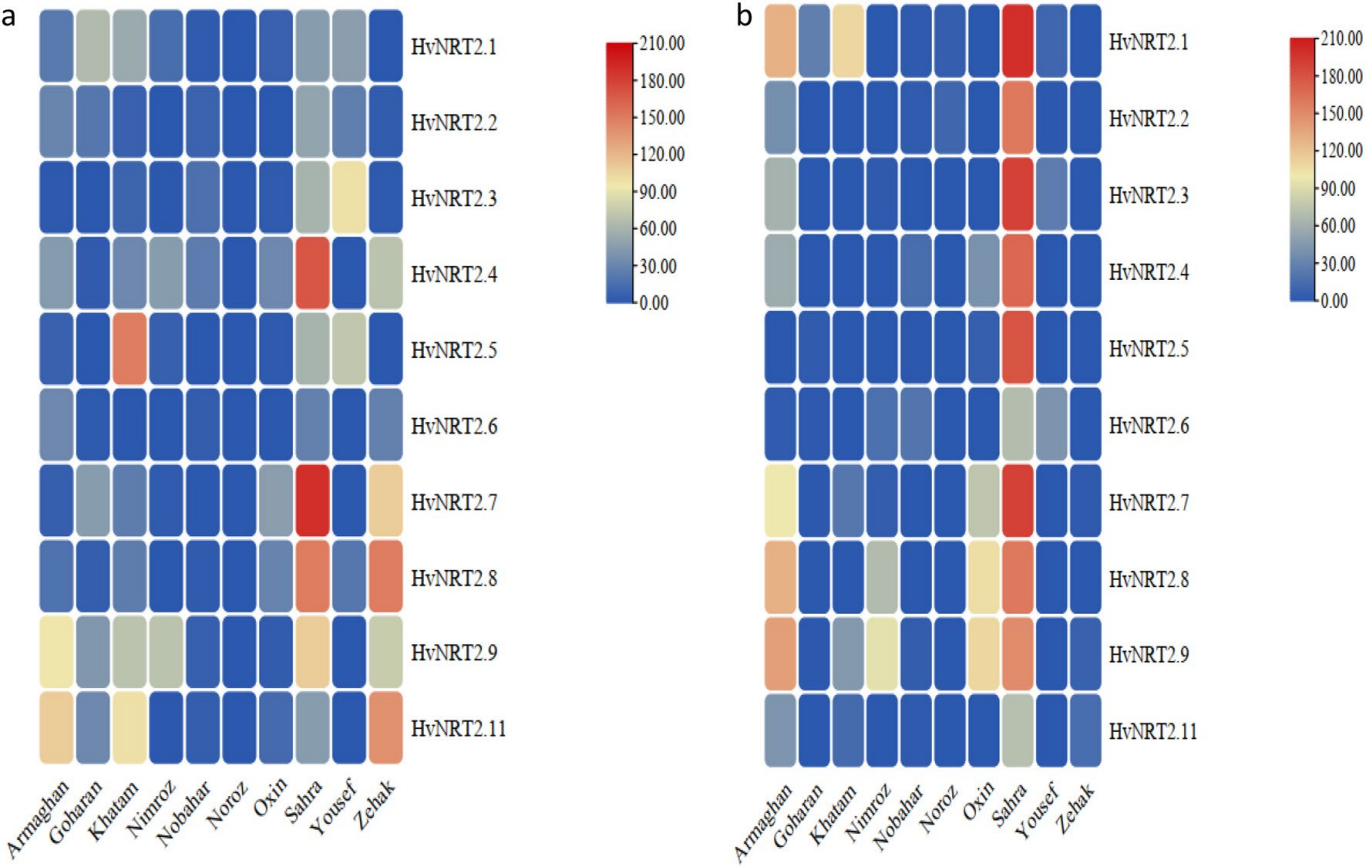
Heatmaps representing the expression profiles of leaf *HvNRT2* genes in response to ND, and their gene expression at 7 (**a**) and 14 (**b**) days after ND application for root. The heat map was generated using TBtools.

**Figure 6:**
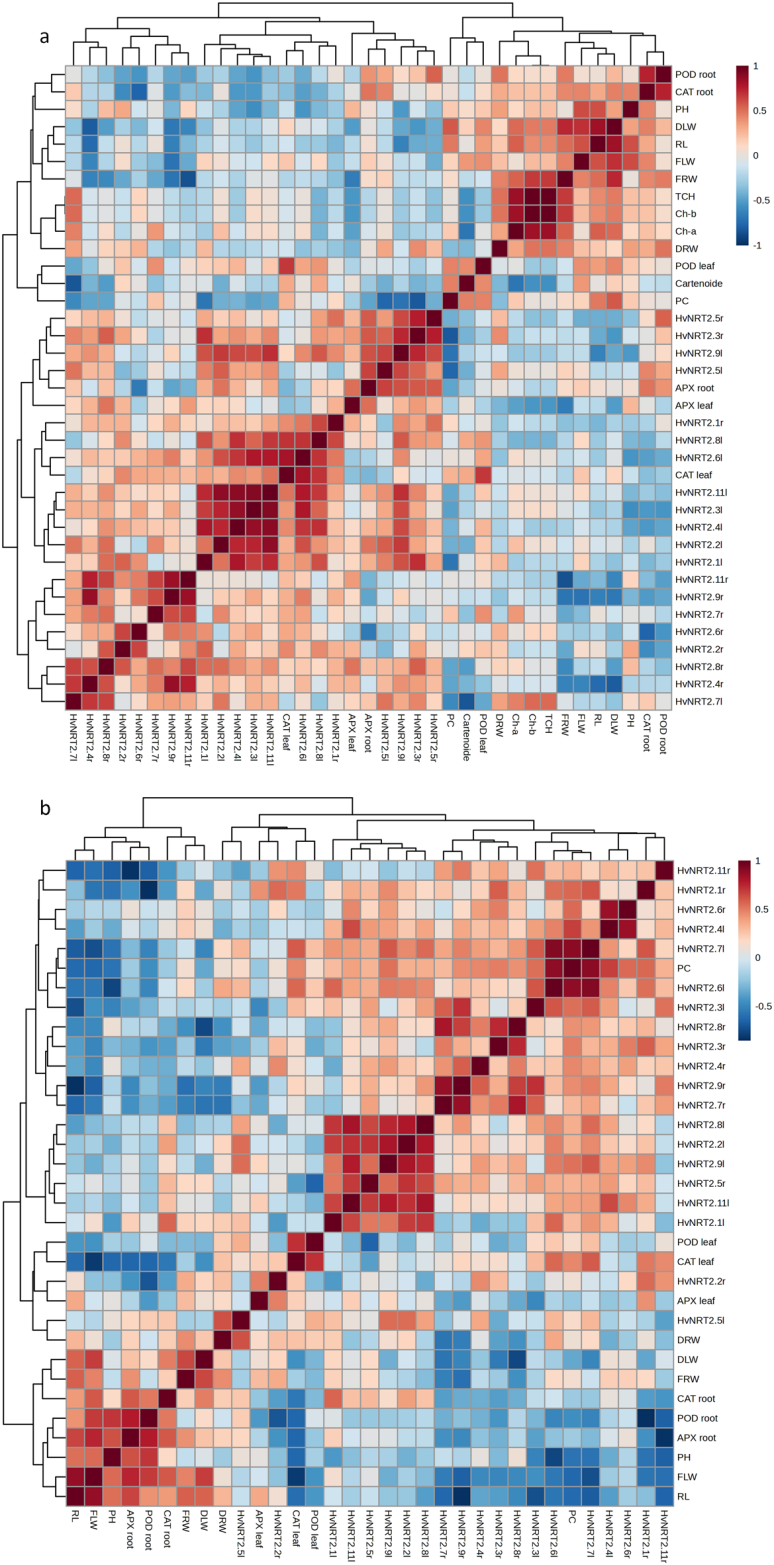
The correlation coefficients of physiological and morphological traits and gene expression at 7 (**a**) and 14 (**b**) days after ND application. Protein content (PC); plant height (PH); leaf dry weight (LDW); leaf fresh weight (LFW); root fresh weight (RFW); root dry weight (RDW); HvNRT2r (root HvNRT2); HvNRT2l (leaf HvNRT2).

The expression profile of the 10 nitrate-transporter genes showed that *HvNRT2.6* and *HvNRT2.11* genes were expressed significantly in the Armaghan cultivar 7 days after applying ND as compared to after 14 days. After 14 days of applying ND, the *HvNRT2.1, HvNRT2.3, HvNRT2.4, HvNRT2.7, HvNRT2.8,* and *HvNRT2.9* genes showed a significantly higher expression than 7 days after the ND application (Fig. 6). In the Goharan cultivar, the expression of *HvNRT2.1, HvNRT2.2, HvNRT2.7, HvNRT2.9,* and *HvNRT2.11* genes increased significantly 7 days after applying ND. The expression of *HvNRT2.4, HvNRT2.5, HvNRT2.8, HvNRT2.9,* and *HvNRT2.11* genes significantly increased in the Khatam cultivar 7 days after applying ND (Fig. 5). After 14 days of applying ND, the *HvNRT2.1* gene expression showed a significant increase as compared to 7 days after applying ND. In the Nimroz cultivar, the *HvNRT2.4* gene expression significantly increased after 7 days as compared to 14 days of applying ND. Two genes, *HvNRT2.8* and *HvNRT2.9,* were significantly expressed after 14 days as compared to 7 days after the ND application. In the Nobahar cultivar*,* the expression of *HvNRT2.3* and *HvNRT2.4* genes rose significantly 7 days after applying ND. After 14 days of applying ND, the *HvNRT2.6* gene showed a significant increase in expression (Fig. 6). In the Noroz cultivar, significant increases in the *HvNRT2.1* and *HvNRT2.2* gene expression were observed 14 days after the ND application. The expression of *HvNRT2.7, HvNRT2.8,* and *HvNRT2.9* genes was redoubled significantly in the Oxin cultivar 14 days after the ND application. In the Sahra cultivar, all *HvNRT2* genes showed a highly significant elevated expression after 14 days as compared to 7 days after the ND application (Fig. 5). In the Yousef cultivar*,* significantly increased expression levels of the *HvNRT2.1, HvNRT2.2, HvNRT2.3, HvNRT2.5,* and *HvNRT2.8* genes occurred 7 days after the ND application. After 14 days of applying ND, only the *HvNRT2.6* gene showed an increase in expression as compared to 7 days after this treatment. In the Zehak cultivar, the expression of *HvNRT2.4, HvNRT2.6, HvNRT2.7, HvNRT2.8, HvNRT2.9,* and *HvNRT2.11* genes rose significantly after 7 days as compared to 14 days after the ND application (Fig. 5).

#### Correlations between the measured traits and gene expression levels 7 and 14 days after the ND condition

##### Correlations between traits 7 days after ND conditions

The *HvNRT2.7l* had a positive correlation with *HvNRT2.8r*. The *HvNRT2.7l* showed a negative correlation with carotenoid content whereas the *HvNRT2.4r* was positively correlated with *HvNRT2.9r* and *HvNRT2.11r*. The Ch-b and TCH had a positive correlation with FRW. The *HvNRT2.9r* and *HvNRT2.11r* showed a negative correlation with FRW, and carotenoid content was negatively correlated with Ch-a content (Fig. 6a).

##### Correlations between the traits 14 days after ND

The RL had positive and highly significant correlations with FLW and APX in roots. The FLW showed positive and significant correlations with APX, POD, CAT, FRW, and DLW in roots. The plant height (PH) had a positive and significant correlation with the root POD. The root APX had a positive and significant correlation with the root POD. The leaf CAT and *HvNRT2.1l* were positively correlated with leaf POD and *HvNRT2.8l*, respectively. *The HvNRT2.11l* was positively correlated with *HvNRT2.9l*, *HvNRT2.2l*, and *HvNRT2.8l*. Positive correlations were observed between *HvNRT2.2l* and *HvNRT2.5r*, *HvNRT2.9l*, and *HvNRT2.8l*. The *HvNRT2.7r* was positively correlated with *HvNRT2.9r* and *HvNRT2.8r*. The *HvNRT2.9r* had positive correlations with *HvNRT2.8r* and *HvNRT2.3l*. The *HvNRT2.6l* showed a positive correlation with PC, and PC was positively correlated with *HvNRT2.4l*. There was a positive correlation between *HvNRT2.4l* and *HvNRT2.6r* (Fig. 6b).

##### Cluster analysis of the cultivars in response to ND

**Figure 7:**
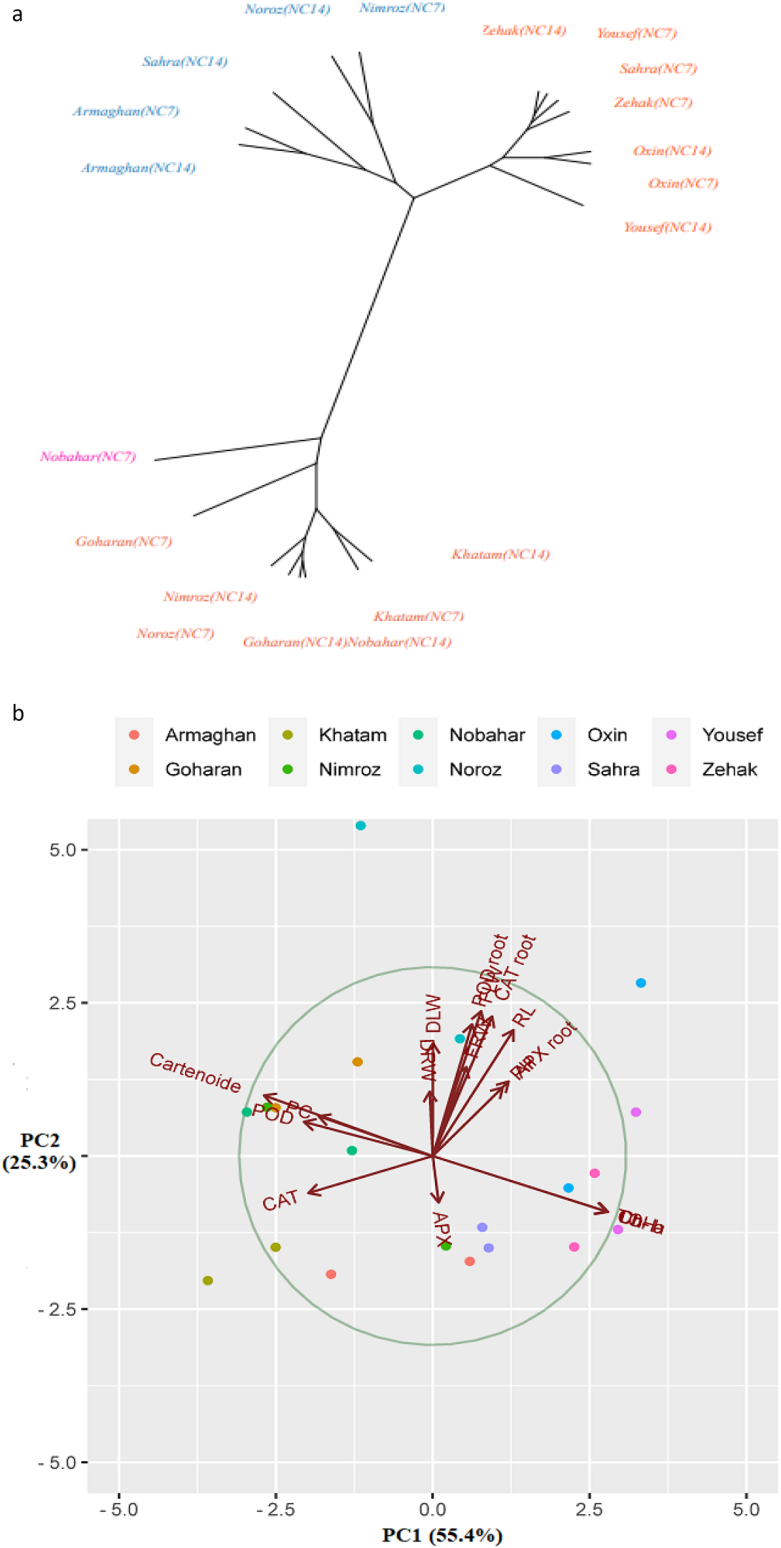
The clustering of cultivars (**a**) and principle component analysis of 10 barley cultivars (**b**).

The cluster analysis of morphological and physiological traits showed that the cultivars were divided into four groups. Tolerant and semi-tolerant cultivars, such as Zehak (NC7 and NC14), Oxin (NC7 and NC14), Sahra (NC7), and Yousef (NC7 and NC14), were placed in the first cluster. The Nobahar (NC7), a sensitive cultivar, was placed in the second cluster. The third cluster included semi-tolerant cultivars, such as Armaghan (NC7 and NC14), Sahra (NC14), Nimroz (NC7), and Noroz (NC14) (Fig. 7a). The fourth cluster included Khatam (NC7 and NC14), Goharan (NC7 and NC14), Nimroz (NC14), Noroz (NC7), and Nobahar (NC14) as sensitive and semi-sensitive cultivars. The diversity of cultivars in all environments for morphological and physiological traits was evaluated using the bi-plot graphic display, and the cultivars were evaluated according to their principal component analysis (PCA) scores. The two principle components determined 80.7% of the variation. The Noroz, Yousef, Oxin, Zehak, and Sahra cultivars are considered tolerant cultivars located on the upper right side of the graph. The Khatam, Nimroz, Nobahar, Armaghan, and Nobahar cultivars are considered sensitive and semi-sensitive cultivars located on the left bottom of the diagram (Fig. 7b).

## Discussion

Nitrogen is one of the main components of amino acids, proteins, and nucleic acids, which are core nutrients for the building blocks of plant cells^20^. Cultivars that use N more efficiently are one of the main objectives of barley breeding programs. The physiological, morphological, and transcriptional analyses were incorporated in this study to obtain insights into the growth of 10 barely cultivars on ND. Sahra revealed a high DRW, protein content, Chl a/b, TCH, APX, CAT, and POD in roots and leaves after ND treatment. Furthermore, the highest *HvNRT2* gene expression was measured in Sahra after the ND application. In the present study, the 10 barely cultivars were significantly different for DRW and leaf chlorophyll content at the seedling stage under ND, suggesting that those two indicators could be used for screening barely cultivars under ND conditions at the seedling stage.

### Plant growth and development traits

Significant differences among the 10 cultivars were detected for FRW, DRW, and FLW. The differences in growth performance and N accumulation between the 10 barely cultivars indicated that Sahra and Yousef could maintain better growth than the other eight cultivars under the LN treatment. The DRW was higher in Sahra than in the other nine cultivars. Based on the previous studies, root development might be related to the ND-induced signaling cascade^21,22^. Root growth was stimulated by both the increased root uptake area and reduced nutrient demand in shoots^23^. In *C. odorata* plants experiencing nitrogen deficiency, metabolic resources were allocated preferentially to root system growth^24^.

### Physiological traits in ND application

On 7 and 14 days after the ND application, significant increases in Chl-a/b, TCH, and antioxidant enzyme content in Sahra, Yousef, and Zehak cultivars were observed as compared to normal treatment; therefore, these are considered ND-tolerant cultivars. An indicator of plant growth and development is photosynthetic capacity^25^. In our study, Sahra, Yousef, and Zehak increased photosynthetic efficiency compared to seven cultivars, indicating a high tolerance to ND. Chlorophylls a/b in leaves were increased in tolerant cultivars (Sahra and Yousef) 7 and 14 days after the application of ND. According to other findings, leaf chlorophylls a/b were increased under ND in wheat^26^ and rice^27^. In previous studies, significant differences in chlorophyll concentrations were found in sorghum, rice, maize, and pearl millet under ND conditions^28^. The main ROS-scavenging enzymes in plants are POD, APX, and CAT. The observed increases in the activities of these enzymes contribute to an increase in AOX activity, especially in plants subjected to ND^29^.

### Gene expression analyses 7 and 14 days after ND conditions

To identify the molecular mechanisms adopted by ND-tolerant barley cultivars under ND conditions, the differences in the expression levels of *HvNRT2* genes involved in nitrogen metabolism were compared between ND-tolerant and ND-sensitive barley cultivars. On 7 and 14 days after ND, Sahra, Zehak, and Yousef showed higher significant expression levels in leaves and roots than the other cultivars. At the seedling stage, *HvNRT2.1, HvNRT2.2, HvNRT2.4,* and *HvNRT2.5* genes were mainly expressed in leaves and roots 7 days after the ND application. The *AtNRT2.1*, *AtNRT2.2*, *AtNRT2.4*, and *AtNRT2.5* play key roles in root NO^-^_3_ uptake in *Arabidopsis*^30^. The potential role of NRT2.1 as a nitrate detector has been suggested due to its strong attraction toward nitrate and its influence on root structure in environments with limited nitrogen^31^. In cotton, the overexpression of *NRT2* genes could increase both nitrate uptake and transport as well as the plant biomass in response to ND^32^. The presence of *NRT2* genes is crucial in enhancing the uptake and transport of N in conditions where it is deficient. *NRT2.4* is believed to facilitate the transportation of N from roots to shoots in response to N deficiency^33^. In addition, while *NRT2.4* and *NRT2.5* genes were predominantly expressed in roots, their presence was also observed in shoots, indicating that these genes play a role in N transport during nitrogen deficiency^34^.

### Correlations and cluster analyses of the 10 cultivars on ND conditions

Based on the data from the 10 barely cultivars and the correlation analysis, the modifications of FLW were closely correlated with DLW, RL, and FRW, demonstrating that root and shoot development could also be considered useful indicators for the evaluation of the plant’s ND response. In our study, the Sahra possessed high FLW, DLW, and FRW as compared with the other cultivars. Cultivars such as Sahra and Yousef showed a significant increase in leaf CAT content. In the tolerant genotypes, antioxidant enzymes showed a significant increase, indicating the high tolerance of these cultivars in response to ND stress in the long term. According to our findings, tolerant cultivars had greater antioxidant enzyme activity in the root and leaf than sensitive accessions. A positive correlation between NUE and root development was observed in rice under low and high nitrogen levels, indicating a relationship between NUE and root growth^35^. The results of the PCA and cluster analysis showed that tolerant and sensitive cultivars were placed in a group with almost a similar pattern under ND stress. The Sahra, Yousef, and Zehak cultivars were nitrogen-tolerant cultivars.

## Conclusion

In the present study, 10 cultivars were exposed to ND application for 7 and 14 days. The *HvNRT2* genes were up-regulated suggesting that the nutrient transporting and antioxidant regulation may play an important role in barely in response to ND. A positive correlation between NUE and root development was observed under ND. Overall, Sahra cultivar showed high biomass and protein content in response to ND stress, making it a good candidate as a tolerant ND cultivar at seedling stage.

## Methods

The plant material was officially obtained from the Seed and Plant Improvement Institute (SPII), formally identified by the corresponding author Dr. Abbas Saidi, and confirmed by Dr. Habibollah Ghazvini in the Cereal Research Department (CRD), at the SPII, which deposits the collected material. Furthermore, the use of plants in the present study complies with all necessary international, national, and institutional guidelines and legislation. All plant material is owned by the authors and no permission is required for their use. Information on the voucher specimen was obtained by Dr. Habibollah Ghazvini. The seeds of 10 barley cultivars were germinated on wet Whatman filter papers in Petri dishes (Table 2). Seven to 10 day-old seedlings with a uniform growth state, were moved to 10-L containers. The plants were treated with a modified Hoagland nutrient solution^36^ containing 2 mmol/L of NH^+^_4_NO^-^_3_, 0.4 mmol/L of MgSO_4_, 0.3 mmol/L of K_2_SO_4_, 0.2 mmol/L of KH_2_PO_4_, 0.4 mmol/L of CaCl_2_, 0.19 µmol/L of CuSO_4_, 46.9 µmol/L of H_3_BO_3_, 4.5 µmol/L of MnCl_2_, 1 µmol/ L of Na_2_MoO_4_, 0.38 µmol/L of ZnSO4, and 19.9 µmol/L of Fe (III) EDTA. The pH of the solution was adjusted to 5.8 with NaOH. The two N treatments included 0.2 mmol/L of NH^+^_4_NO^-^_3_ (ND) and 2 mmol/L NH^+^_4_NO^-^_3_ (as a control). Plant height (PH), dry leaf weight (DLW), dry root weight (DRW), fresh leaf weight (FLW), fresh root weight (FRW), and root length (RL) were recorded after 7 and 14 days of N treatments. Plants with a uniform growth status were subsequently harvested as replicates, separated into roots and shoots, and dried in an oven at 72 °C for 3 days to obtain DRW and DLW.

**Table 2:** Characteristics of barley cultivars used in this study.

Cultivars	Pedigree	Origin	Internet access	Row type	Growth habit	Adaptability to climate zone
Sahra	LB.Iran/Una8271//Gloria"S"/Com"S"	CIMMYT-ICARDA	http://spii.ir/_DouranPortal/Documents/sahrajo_20170129_144252.pdf	6-rowed	Facultative	Warm and humid zone (North of Iran)
Nowruz	GOB/Aleli//Canela/3/Arupo*2/Jet/4/Arupo/K8755//Mora (WB-90-15)	CIMMYT-ICARDA	http://spii.ir/_DouranPortal/Documents/jo.nourooz.96_20190728_152953.pdf	2-rowed	Spring	Warm and dry zone (South of Iran)
Armaghan	Legia//Rhn/Lignee 527	Iran	http://spii.ir/_DouranPortal/Documents/armaghan_20180613_102621.pdf	6-rowed	Spring	Temperate zone (Central parts of Iran)
Yousef	Lignee 527/Chn-01//Gustoe/4/Rhn-08/3/Deir Alla 106//Dl71/Strain 205	ICARDA	http://spii.ir/_DouranPortal/Documents/yosefjo_20170129_144753.pdf	6-rowed	Spring	Temperate zone (Central parts of Iran)
Nobahar	GOB/Aleli//Canela/3/Arupo*2/Jet/4/Arupo/K8755//Mora (WB-90-14)	CIMMYT-ICARDA	http://spii.ir/_DouranPortal/Documents/%D9%86%D9%88%D8%A8%D9%87%D8%A7%D8%B1_20230902_122720.pdf	2-rowed	Spring	Warm and humid zone (North of Iran)
Oxin	Rojo/Sahra	Iran	http://spii.ir/_DouranPortal/Documents/oxin_20180613_102706.pdf	6-rowed	Facultative	Warm zone (North and south of Iran)
Zahak	Poa/Hjo//Qjina	CIMMYT-ICARDA	http://spii.ir/_DouranPortal/Documents/zahakjo_20170129_144856.pdf	6-rowed	Spring	Warm and dry zone (South of Iran)
Nimrooz	Trompillo	CIMMYT-ICARDA	http://spii.ir/_DouranPortal/Documents/nimrozjo_20170129_143906.pdf	2-rowed	Spring	Warm and dry zone (South of Iran)
Khatam	Sahra/Kavir	Iran	http://spii.ir/_DouranPortal/Documents/khatam_20180617_112452.pdf	6-rowed	Facultative	Temperate zone (Central parts of Iran)
Goharan	Rhn- 03//L.527/NK1272	ICARDA	http://spii.ir/_DouranPortal/Documents/goharan_20180617_112407.pdf	6-rowed	Spring	Temperate zone (Central parts of Iran)

## Characterization of physiological traits

To investigate the physiological traits, normal and stressed barley leaves and roots were collected at the two-week seedling stage 7 and 14 days after the application ND. Fresh leaf samples were washed with distilled water in the laboratory and then left to dry at room temperature (18 °C) for 6 h for the analysis of chlorophylls (Chl-a and Chl-b) and carotenoid contents. An accurately weighed (0.5 g) fresh plant leaf sample was homogenized in a tissue homogenizer with 10 ml of acetone as the extraction solvent. The homogenized sample was centrifuged at 12,000 rpm at 4 °C for 15 min. One ml of the separated supernatant was mixed with 4 ml of the acetone solvent. The solution mixture was analyzed for Chl-a, Chl-b, total chlorophyll, and carotenoid contents by spectrophotometry using the following Equations^37^:

$$ \begin{aligned} & {\text{Ch - a}} = {12}.{\text{25A}}_{{{663}.{2}}} {-}{\text{ 279A}}_{{{646}.{8}}} \\ & {\text{Ch - b}} = {21}.{\text{5A}}_{{{646}.{8}}} {-}{ 5}.{\text{1A}}_{{{663}.{2}}} \\ & {\text{C}}_{{{\text{x}} + {\text{c}}}} = \, \left( {{1}000{\text{A}}_{{{47}0}} {-}{ 1}.{\text{82C}}_{{\text{a}}} {-}{85}.0{\text{2C}}_{{\text{b}}} } \right)/{198} \\ & {\text{Total chlorophyll}} \left( {{\text{mg}}/{\text{g}}} \right) \, = \, \left[ {{2}0.{2 }\left( {{\text{A}}_{{{645}}} } \right) \, + {8}.0{2 }\left( {{\text{A}}_{{{663}}} } \right)} \right] \\ \end{aligned} $$

A = Absorbance, Chl-a = Chlorophyll a, Chl-b = Chlorophyll b, Cx + c = Carotenoids.

## Determination of antioxidant enzyme activity

Fresh root and leaf tissues (0.5 g) were ground into fine powder under liquid nitrogen and then mixed with 10 mL of pre-cooled phosphate buffer (50 mM, pH 7.8) containing 1.0% (w/v) PVP. The mixture was centrifuged at 8000 × g and 4 °C for 40 min. The obtained supernatant was used for the enzyme assay as crude enzyme preparation. Catalase (CAT)^38^, ascorbate peroxidase (APX)^39^, and peroxidase (POD)^40^ activities were assayed according to Ekinci et al., Nakano and Asada, and Chance and Machly, respectively. Since the addition of H_2_O_2_, changes in absorbance were monitored for 120 s at 240, 290, and 470 nm to measure CAT, APX, and POD activities, respectively.

## Protein extraction

Protein and enzyme extracts were prepared following the previous method^41^. In brief, fresh leaves (0.5 g) were ground to a fine powder in liquid nitrogen using a pre-chilled mortar and pestle and then extracted in 3 mL of 0.2 M potassium phosphate buffer (pH 7.0), containing 0.1 mM of ethylenediaminetetraacetic acid (EDTA). The extract was centrifuged at 13,000 rpm and 4 °C for 20 min, and the supernatant was used for the protein activity assay. The total protein content for all samples was determined by the method of Bradford (1976) using bovine serum albumin as a standard^42^.

## RNA extraction and expression patterns of nitrate-transporter genes

To prepare leaf RNA, leaves and root samples were collected separately from barley seedlings under ND stress (on 7 and 14 days) and normal conditions. Total RNA was extracted from nitrogen-stressed and normal leaves and roots using an RNX-Plus kit (Sinaclone) according to the manufacturer's instructions. The purity and concentration of RNA were determined by NanoDrop, and its quality was confirmed using the 1% agarose gel analysis. Then, cDNA was synthesized according to the instructions of a cDNA synthesis kit. Each gene was analyzed with three repetitions, where the actin gene was used as a reference gene. All primers used in the gene expression analysis were designed using the Oligo program (Table 3). Gene expression was examined with a real-time instrument using Cybergreen as described in the manufacturer's instructions. After normalization, the relative expression of genes was evaluated through 2^-∆∆CT^ and the value of Ct for nitrate transporter genes was determined using actin as a reference gene. The qRT-PCR analysis was performed to determine the expression profiles of *HvNRT2.1, HvNRT2.2, HvNRT2.3, HvNRT2.4, HvNRT2.5, HvNRT2.6, HvNRT2.8, HvNRT2.9*, and *HvNRT2.10* genes using leaf and root tissues under normal and ND treatments. The expression levels of these genes were also investigated at the seedling stage.

**Table 3:** The primer sequences of *HvNRT2* genes used in this study.

Primer name	Primer sequence
HvNRT2.1F	F: GCTCCGCATGTTGATGTTTA
HvNRT2.1R	R: TGACGTTGCCGTTTGACTTA
HvNRT2.2F	TCATCTGTCTGCAGGAATCG
HvNRT2.2R	CCACATGTAACTGCGCGTAT
HvNRT2.3F	CATATCGCAGGCCAAAAGTT
HvNRT2.3R	CCTTATACGTGCTGGGGTGT
HvNRT2.4F	CCAGCACGTATGAGACTGGA
HvNRT2.4R	ACGCCTTATTACAGCCGATG
HvNRT2.5F	TGGACCGAGGAGGAGCGT
HvNRT2.5R	GGAGCTCTTCGGACCTCACAC
HvNRT2.6F	CATGCACGCTGCCCGTCGCT
HvNRT2.6R	GTCTCATACGTGCTGGGGCG
HvNRT2.7F	F: AGTACTACGGTGCCGAGTGG
HvNRT2.7R	R: GTTTGGTGGGCTGGTAGGTA
HvNRT2.8F	GACCGAGGAGGACTACTACGC
HvNRT2.8R	CGTGTACGGTAGGGAAGTAG
HvNRT2.9F	TCCATGCTCCTCCCACCCA
HvNRT2.9R	CGTGTACGGTAGGGAAGTAG
HvNRT2.10F	CTGCATCAGGGCTTACCTTC
HvNRT2.10R	CGGAGGGAGTAGGTTGGTAAA
HvActinF	F: GGTCCATCCTAGCCTCACTC
HvActinR	R: GATAACAGCAGTGGAGCGCT

## Statistical analysis

The TB tools were utilized to draw the heat map, which was used to display the differential expression of genes and the correlation between the physiological traits and gene expression. Statistical analyses were performed using SPSS version 20.0 statistical software. Significant variations between means were compared at *P* < 0.05 (Duncan's test). Statistical graphs were generated using GraphPad Prism version 9 software at a statistical significance of *p* < 0.05.

## Data Availability

All data generated or analyzed during this study are included in this present article**.**
